# Heterologous SH3-p85β inhibits influenza A virus replication

**DOI:** 10.1186/1743-422X-7-170

**Published:** 2010-07-23

**Authors:** Dan-gui Zhang, Wei-zhong Li, Ge-fei Wang, Yun Su, Jun Zeng, Chi Zhang, Xiang-xing Zeng, Xiao-xuan Chen, Yan-xuan Xu, Kang-sheng Li

**Affiliations:** 1 Department of Microbiology and Immunology, Key Immunopathology Laboratory of Guangdong Province, Shantou University Medical College, 22 Xinling Road, Shantou, 515041, China

## Abstract

Phosphatidylinositol 3-kinase (PI3K)/Akt signalling pathway can support the replication of influenza A virus through binding of viral NS1 protein to the Src homology 3 (SH3) domain of p85β regulatory subunit of PI3K. Here we investigated the effect of heterologously overexpressed SH3 on the replication of different influenza A virus subtypes/strains, and on the phosphorylation of Akt in the virus-infected cells. We found that heterologous SH3 reduced replication of influenza A viruses at varying degrees in a subtype/strain-dependent manner and SH3 overexpression reduced the induction of the phosphorylation of Akt in the cells infected with PR8(H1N1) and ST364(H3N2), but not with ST1233(H1N1), Ph2246(H9N2), and Qa199(H9N2). Our results suggest that interference with the NS1-p85β interaction by heterologous SH3 can be served as a useful antiviral strategy against influenza A virus infection.

## Background

Influenza A viruses are globally important human and animal respiratory pathogens, and viral infections cause highly contagious respiratory diseases. Influenza A virus can be divided into numerous subtypes (H1~H16 and N1~N9) according to the antigenicity of hemagglutinin (HA) and neuraminidase (NA). Among them, H1N1 and H3N2 subtypes are the most common subtype in human influenza infections [1]. However, in some situations, several avian influenza virus subtypes (such as H5N1, H7N7, or H9N2) can break through the species barrier and be transmitted to humans [2]. These avian influenza viruses have posed serious threat to public health.

One of the main research emphases in the influenza A virus is its NS1 protein. NS1 can modulate virus infection and host cell signalling pathway [3-6], such as phosphatidylinositol 3-kinase(PI3K)/Akt pathway [7]. The PI3K/Akt pathway plays a central role in modulating diverse downstream signalling pathways associated with cell survival, proliferation, migration, and differentiation [8-10].

PI3K is a dimeric enzyme consisting of a p110 catalytic subunit (α, β, or δ) tethered to a smaller, non-catalytic, regulatory subunit p85 (usually p85α, p85β, p55γ, p55α, or p50α) [11-13]. NS1 can interact with p85β of PI3K via direct binding to SH3 domain of p85β and hence promote the activation of PI3K [14], whereas mutation within the SH3 binding motif 1 of NS1 is able to deprive NS1-p85β interaction and result in the reduction of virulence of influenza A virus [15]. Apart from SH3, iSH2 domain (inter-SH2) and cSH2 domain (C-terminal SH2) domain of p85β are responsible for NS1-p85β interaction and the subsequent activation of PI3K [14,16,17]. NS1-mediated PI3K activation is obviously essential for influenza A virus replication because viral titers are significantly decreased when PI3K is inhibited [18,19].

We hypothesized that viral replication could be repressed by blocking NS1-p85β interaction (competitively) with heterologously expressed SH3. Since previous studies have shown that NS1-mediated PI3K activation is obviously important for the efficient propagation of influenza A virus [7,14,18,20,21], in this study, we examined the effect of heterologous SH3 (h-SH3) on (i) the replicability of five strains of influenza A virus from three subtypes (H1N1, H3N2, and H9N2) in infected cells and (ii) the phosphorylation status of Akt after viral infection.

## Results

### Co-localization of h-SH3 and NS1

**Figure 1:**
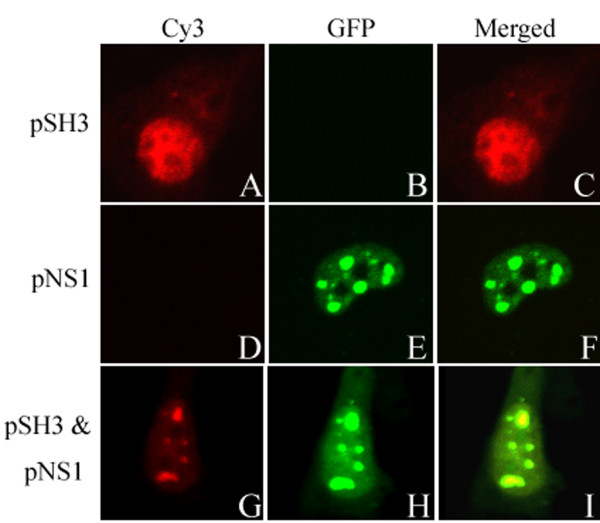
**Confocal imaging of MDCK cells over-expressing SH3 and NS1**. pSH3 (SH3-expressing plasmid based on a modified pcDNA3 vector with the N-terminal Flag) and pNS1 (NS1-expressing plasmid with the pEGFP-c1 backbone) were transiently transfected into MDCK cells individually or together. 24 h post-transfection, cells were stained with anti-Flag antibody and Cy3-labeled secondary antibody. Detection of Cy3 channel seen as red (A, D, and G), GFP channel seen as Green (B, E, and H), and Merged these two channels (C, F, and I).

Confocal microscopy was used to monitor the MDCK cells transiently expressing h-SH3, NS1 (NS11, NS32, or NS92), and co-expressing h-SH3 and NS1 (NS11, NS32, or NS92). We found that h-SH3 mainly presented diffused distribution within the nucleus and lesser within the cytoplasm (Fig. 1A). And NS1 mainly presented diffused distribution or some dot-like structures within the nucleus (Fig. 1E). When co-expression, we found that h-SH3 and NS1 mainly presented diffused distribution or some dot-like structures within the nucleus (Fig. 1G and 1H), and h-SH3 located at the same position as NS1 which suggested the interaction between h-SH3 and NS1 (Fig. 1I). Since NS1 from different influenza A virus strains (NS11, NS32, or NS92) displayed the similar co-localized pattern with h-SH3 in MDCK cells, only a representative figure was provided.

### Differential suppressive effect of h-SH3 on the replication kinetics of influenza A viruses

**Figure 2:**
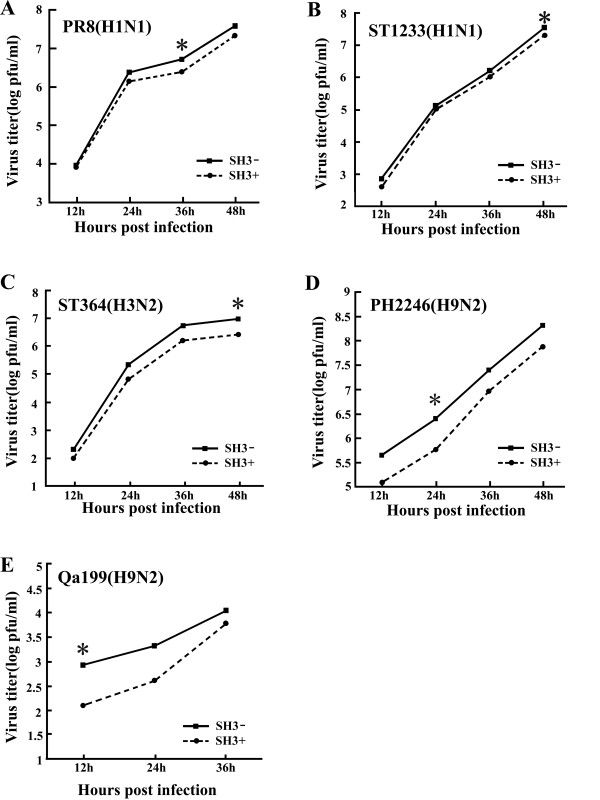
**Replication kinetics of influenza A viruses in MDCK(SH3+) and control MDCK(SH3-) cells**. Cells were infected with influenza A viruses at an MOI of 0.001 and incubated with serum-free medium containing 1 ug/ml TPCK-trypsin. The yield of virus in the culture supernatant was titrated every 12 h, from 12 h post-infection, by plaque assay in MDCK cells. The time point where maximal reduction was observed is indicated with asterisk (*).

To examine if the replication of influenza A viruses could be affected by h-SH3, we examined viral titers in MDCK(SH3+) cells (MDCK cells transfected with SH3-expressing plasmid) and control MDCK(SH3-) cells (MDCK cells transfected with empty vector). Cells were infected with a relatively low dose of influenza A viruses (MOI = 0.001) and virus yield was determined by plaque assay in MDCK cells. In comparison with the control, the MDCK(SH3+) showed variable degrees of reduction in viral titers at different time points (Fig. 2). The maximal reduction observed was about 8-fold with ST364, about 6-fold with Qa199, about 4-fold with Ph2246, and about 2-fold with ST1233 and PR8. These findings indicate differential sensitivities of influenza A virus subtypes/strains to h-SH3.

### Differential suppressive effect of h-SH3 on the phosphorylation status of Akt

**Figure 3:**
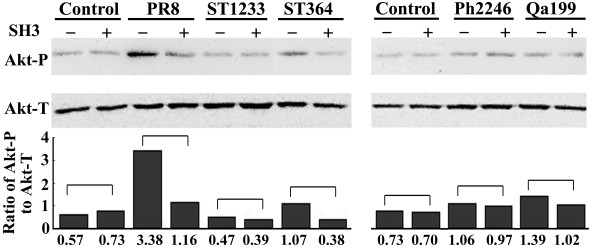
**Western blot analysis of phosphorylated Akt level in MDCK cells**. MDCK cells with or without heterologous SH3 were infected with PR8, ST1233, ST364, Ph2246, or Qa199 at an MOI of 2 for 8 h. Akt-P (phosphorylated Akt at Serine 473) and Akt-T (total Akt) in cellular lysate were detected using anti-Akt-P and anti-Akt-T antibodies, respectively, and peroxidase-conjugated secondary antibody.

As the h-SH3 had differential suppressive effect on the replication of influenza A viruses, we further examined the phosphorylation status of Akt (Akt-P) at Ser-473, which is the marker commonly used to monitor the activation state of PI3K [22], by measuring the ratio of phosphorylated Akt (Akt-P)/total Akt (Akt-T) in virus-infected MDCK(SH3+) and MDCK(SH3-) cells. Western blot analysis showed that, compared to the uninfected MDCK(SH3-) cells, the Akt-P level increased in PR8-, ST364-, Ph2246-, or Qa199-infected MDCK(SH3-) cells but not in ST1233-infected MDCK(SH3-) cells. Moreover, we observed the similar Akt-P and Akt-T levels in MDCK(SH3+) and MDCK(SH3-) cells. In addition, we found no change of Akt-P/Akt-T ratio in the ST1233-, Ph2246-, or Qa199-infected MDCK(SH3+) cells, but significant reduction of Akt-P/Akt-T ratio in the PR8- and ST364-infected MDCK(SH3+) cells (Fig. 3). These data suggested that in the absence of viral infection, h-SH3 had no effect on the phosphorylation of Akt and that the suppressive effect of heterologous SH3 on the phosphorylation status of Akt was viral strain-specific.

### Differential roles of PI3K in the replication of ST364 and PR8 viruses

Having demonstrated the suppressive effect of h-SH3 on the phosphorylation status of Akt in the ST364(H3N2)- and PR8(H1N1)-infected cells, we further examined the role of PI3K in the replication of these two viral strains by treating them with the PI3K inhibitor, LY294002 (10 uM or 50 uM), followed by the plaque formation assay. Significant reduction in the number of plaques was observed in the ST364-infected cells, but there was no obvious change with the PR8-infected cells. Treatment with different concentrations (10 uM and 50 uM) of LY294002 showed no obvious difference in replication.

## Discussion

The binding of p85β subunit to the influenza A virus NS1 protein results in the activation of PI3K/Akt pathway, which in turn can promote the viral replication by helping virus entry, viral RNA expression, nuclear export of the RNPs, and preventing premature apoptosis [19,23]. As NS1 interacts with p85β via direct binding to SH3, iSH2, or cSH2 domain of p85β, it can be speculated that disturb this interaction by heterologously expressed SH3, iSH2, or cSH2 may be a potential anti-influenza strategy. Since iSH2 is also an interactive site of p85β and p110 [16,17], overexpression of iSH2 may interfere with the formation of PI3K in normal cells. Additionally, given that the relative weak ability of cSH2 in mediating the interaction of p85β and NS1 [14], only SH3 was thus chosen as a candidate antiviral agent in this study. Our aim was to determine whether the SH3-p85β site in the NS1 could be targeted by heterologously expressed SH3, thereby inhibiting influenza A virus replication.

Subcellular localization in this study showed that in MDCK cells transiently co-expressing NS11, NS32, or NS92, the h-SH3 was colocalized with NS1 in the nucleus (Fig. 1), implying the interaction of NS1 and h-SH3. Combined with the results of Fig. 2, it can be seen that in addition to its recognized interaction with endogenous p85β subunit of PI3K via three binding sites (SH3, iSH2, and cSH2) [14,16,17], NS1 can also bind to heterologous SH3 and the interaction is strong enough to result in suppression of viral replication, though the degree of suppression was apparently different among the viral strains.

As Akt-P is the major downstream product of activated PI3K, the Akt-P/Akt-T ratio was used to compare the levels of virus-induced PI3K activation affected by h-SH3 in cells infected with different influenza A viruses. Remarkable elevation of Akt-P could be seen in PR8-, ST364-, Ph2246-, or Qa199-infected cells, but not in ST1233-infected cells. In addition, h-SH3 could reduce the PI3K activation in PR8- or ST364-infected cells, while no alteration was observed in ST1233-, Ph2246-, or Qa199-infected cells (Fig. 3). The reason for this is unclear. As iSH2 domain may be more important than SH3 for NS1-p85β interaction [16,17], it is reasonable to speculate that for some influenza A virus strains, h-SH3 could not be absolutely essential for the activation of PI3K. Thus, even through h-SH3 can disturb the NS1-p85β interaction, its effect on the activation of PI3K and the ratio of Akt-P/Akt-T may be negligible.

It has been shown that the PI3K/Akt pathway activated by NS1 is beneficial for influenza A virus infection [22], and influenza A virus titers are significantly decreased when PI3K is inhibited [18]. Interestingly, h-SH3's effect on PI3K/Akt pathway was not well correlated with the replication of some viruses in this study. While h-SH3 could inhibit replication of every strain of influenza A virus to varying degrees, no suppression of PI3K (or PI3K/Akt pathway) was observed in ST1233-, Ph2246-, or Qa199-infected MDCK cells (Fig. 3). The reason may involve the complicated relationship between NS1 and cellular proteins. More than nine cellular proteins were found directly interacted with NS1. Among them, CPSF30 (30 kDa subunit of cleavage and polyadenylation specificity factor) and p85β shared partial overlapped binding sites (SH3 binding motif 1) in NS1 [24]. Moreover, blocking the binding of endogenous CPSF30 to NS1 protein can strikingly inhibit the replication of influenza A virus [25]. Therefore, h-SH3 may also disrupt the interaction of NS1 and CPSF30 and then reduce the replication of some influenza A viruses (such as ST1233, Ph2246, and Qa199) without the reduction of Akt-P/Akt-T ratio.

**Figure 4:**
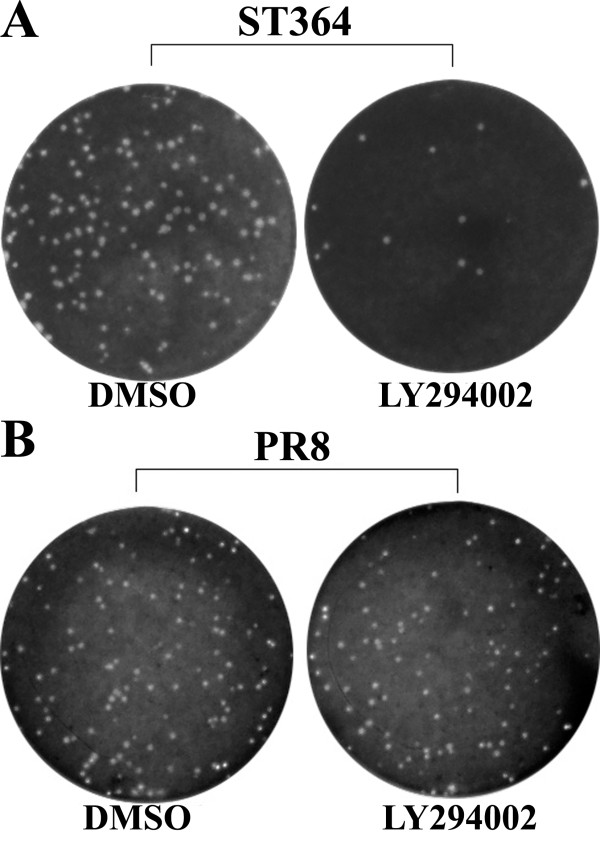
**Differential roles of PI3K in replication of ST364(A) and PR8(B) viruses**. MDCK cells treated with LY294002 (10 uM) for 2 h and MDCK cells treated with DMSO (control) were infected with ST364(H3N2) or PR8(H1N1) viruses and were subjected to the plaque assay.

Of note, the inhibitory effect of h-SH3 was stronger to ST364 virus than PR8 virus. This finding is not consistent with the similar reduction in the Akt-P/Akt-T ratio in PR8-infected cells and ST364-infected cells. One possible explanation is that influenza A virus varied in their sensitivity to PI3K/Akt pathway due to long-time evolution. And we found that compared to ST364 virus, PR8 virus was less susceptible to the treatment of LY294002 (PI3K inhibitor), which suggested that the activated PI3K/Akt pathway is more important for ST364 replication than PR8 replication (Fig. 4).

## Conclusions

In conclusion, this study demonstrates that h-SH3 can inhibit the replication of influenza A viruses (H1N1, H3N2, and H9N2) at different degrees, and targeting NS1-p85β interaction (PI3K/Akt pathway) or other virus-host interaction may be an attractive strategy against the infection of various influenza A virus subtypes/strains.

## Methods

### Cell lines, viruses, plasmids, and reagents

Madin-Darby canine kidney (MDCK) cell lines were routinely cultured in Dulbecco's modified Eagle's medium (DMEM) supplemented with 10% fetal calf serum and antibiotics (100 U penicillin and 100 ug/ml streptomycin) at 37℃ in 5% CO_2_.

Influenza A virus strains A/PR/8/34(H1N1), A/ST/1233/2006(H1N1), A/ST/364/2005(H3N2), A/Ph/ST/2246/2006(H9N2), and A/Qa/ST/199/2006(H9N2), abbreviated herein to PR8, ST1233, ST364, Ph2246, and Qa199, respectively, were used in this study.

SH3 domain (9-79aa) of p85β subunit was cloned into PNF vector (a modified pcDNA3 plasmid with an N-terminal Flag tag) at the *Bam*HI and *Eco*RI sites to generate pSH3. Full-length NS1 sequence of H1N1, H3N2, or H9N2 viruses was cloned into pEGFP-c1 at the *Bam*HI site to generate pNS11, pNS32, or pNS92 plasmids, respectively. The constructs were verified by DNA sequencing.

Rabbit monoclonal anti-phospho-Akt (Ser473) antibody were purchased from Cell Signaling Technology (Danvers, MA, USA), Rabbit monoclonal anti-Flag antibody from Sigma (St. Louis, MO, USA), Rabbit monoclonal anti-Akt antibody, Cy3-labeled goat anti-rabbit antibody, peroxidase-conjugated goat anti-rabbit antibody, and LY294002 (PI3K inhibitor) from Beyotime Biotechnology (Jiangsu, China), and Lipofectamine 2000 from Invitrogen (Carlsbad, CA, USA).

### Co-localization analysis by confocal microscopy

MDCK cell cultures on the glass coverslips were transfected with indicated plasmids. 24 h post-transfection, the cells were washed twice with phosphate-buffered saline (PBS), fixed for 10 min with 4% paraformaldehyde, permeabilized with 0.2% Triton X-100 for 7 min, and incubated for 30 min in PBS containing 3% Bovine Serum Albumin (BSA). Cells were subsequently incubated at room temperature with anti-Flag antibody (1/500) for 2 h and Cy3-labeled secondary antibody (1/300) for 1 h. Images were captured with the Olympus confocal microscopy.

### Transient transfection, viral infection, and determination of antiviral activity

MDCK cells were seeded onto six-well plates and transfected with pSH3 or PNF empty vector at 60% confluence as previously described [26]. After 48 h of transfection, MDCK cells were infected with influenza A viruses at a multiplicity of infection (MOI) of 0.001 in serum-free DMEM containing 1 ug/ml TPCK-trypsin (Worthington, Freehold, NJ, USA) and incubated at 37°C. An aliquot of the supernatant was harvested every 12 h and virus yield was titrated by plaque assay in MDCK cells.

### Western blot-based phosphorylation assay

MDCK cells transfected with indicated plasmids for 24 h were infected with different influenza A virus strains at an MOI of 2. At 8 h post infection the cells were lysed with Laemmli sample buffer containing 5 mM of NaF for 5 min in boiling water followed by brief sonication. Protein concentration was determined using an RC-DC kit (Bio-Rad, Hercules, CA, USA). Proteins (20 ug/lane) were separated in 10% SDS-PAGE and transferred onto a nitrocellulose membrane. The membrane was blocked with Tris-buffered saline containing 0.1% Tween 20 (TBST) with 10% non-fat milk for 2 h and incubated overnight at 4°C with anti-phospho-Akt (Ser473) (1/500) or anti-Akt (1/700) antibody. The membrane was rinsed extensively in TBST and incubated for 2 h with peroxidase-conjugated secondary antibody (1/1000). Immunoblots were developed using the Enhanced chemiluminescence (ECL) reagents (Pierce, Rockford, IL, USA). Quantity One (Bio-Rad) software was used to analyze images.

### Determination of PI3K effect on the replication of ST364 and PR8 viruses

MDCK cells with 100% confluence were pretreated with 10 uM or 50 uM LY294002 (a specific PI3K inhibitor). After 2 h, MDCK cells were infected with ST364 virus or PR8 virus, and plaque assay was performed.

## Competing interests

The authors declare that they have no competing interests.

## Authors' contributions

Conceived and designed the experiments: KSL, DGZ, WZL, GFW. Performed the experiments: DGZ, WZL, GFW, CZ, XXC, YXX. Analyzed the data: DGZ, WZL, KSL, GFW, YS, XXZ. Wrote the paper: DGZ, WZL, KSL. All authors read and approved the final manuscript.
